# Effects of Curcumin on Glycemic Control and Lipid Profile in Polycystic Ovary Syndrome: Systematic Review with Meta-Analysis and Trial Sequential Analysis

**DOI:** 10.3390/nu13020684

**Published:** 2021-02-21

**Authors:** Yung-Jiun Chien, Chun-Yu Chang, Meng-Yu Wu, Chih-Hao Chen, Yi-Shiung Horng, Hsin-Chi Wu

**Affiliations:** 1 Department of Physical Medicine and Rehabilitation, Taipei Tzu Chi Hospital, Buddhist Tzu Chi Medical Foundation, New Taipei City 231, Taiwan; jessica.kan.48@gmail.com; 2 School of Medicine, Tzu Chi University, Hualien 970, Taiwan; paulchang1231@gmail.com (C.-Y.C.); skyshangrila@gmail.com (M.-Y.W.); 3 Department of Anesthesiology, Taipei Tzu Chi Hospital, Buddhist Tzu Chi Medical Foundation, New Taipei City 231, Taiwan; 4 Department of Emergency Medicine, Taipei Tzu Chi Hospital, Buddhist Tzu Chi Medical Foundation, New Taipei City 231, Taiwan; 5 Department of Otolaryngology-Head and Neck Surgery, Taipei Veterans General Hospital, Taipei 112, Taiwan; michaelchen808@gmail.com

**Keywords:** cholesterol, curcumin, insulin resistance, meta-analysis, polycystic ovary syndrome, trial sequential analysis

## Abstract

The therapeutic effects of curcumin for polycystic ovary syndrome (PCOS) remain inconclusive. The present study aims to evaluate the effects of curcumin on glycemic control and lipid profile in patients with PCOS. PubMed, Embase, Scopus, Web of Science, and Cochrane Library were searched from the inception through 28 November 2020. Randomized control trials (RCTs), which enrolled adult patients with PCOS, compared curcumin with placebo regarding the glycemic control and lipid profile, and reported sufficient information for performing meta-analysis, were included. Three RCTs were included. Curcumin significantly improves fasting glucose (mean difference (MD): −2.77, 95% confidence interval (CI): −4.16 to −1.38), fasting insulin (MD: −1.33, 95% CI: −2.18 to −0.49), Homeostasis Model Assessment of Insulin Resistance (HOMA-IR) (MD: −0.32, 95% CI: −0.52 to −0.12), and quantitative insulin sensitivity check index (QUICKI) (MD: 0.010, 95% CI: 0.003–0.018). It also significantly improves high-density lipoprotein (MD: 1.92, 95% CI: 0.33–3.51) and total cholesterol (MD: −12.45, 95% CI: −22.05 to −2.85). In contrast, there is no statistically significant difference in the improvement in low-density lipoprotein (MD: −6.02, 95% CI: −26.66 to 14.62) and triglyceride (MD: 8.22, 95% CI: −26.10 to 42.53) between curcumin and placebo. The results of the fasting glucose, fasting insulin, HOMA-IR, QUICKI, and total cholesterol are conclusive as indicated by the trial sequential analysis. Curcumin may improve glycemic control and lipid metabolism in patients with PCOS and metabolic abnormality without significant adverse effects. Further studies are advocated to investigate the potential effects of curcumin on hyperandrogenism.

## 1. Introduction

Polycystic ovary syndrome (PCOS) is the most common endocrine disorder in women of reproductive age, with prevalence up to 10% to 16% [1,2]. It is characterized by hyperandrogenism, ovulatory dysfunction, and polycystic ovaries [3]. In addition, nearly half of adult patients with PCOS develop metabolic syndrome and insulin resistance [4,5], and are associated with considerably higher risks of type 2 diabetes mellitus, cardiovascular disease, and even cancer [6,7].

The pathophysiology of PCOS is complex and is believed to involve functional ovarian hyperandrogenism caused by hyperresponsiveness to the stimulation of luteinizing hormone and failed downregulation of thecal androgen production [8,9]. A distinctive feature of PCOS is insulin-resistant hyperinsulinemia. It aggravates hyperandrogenism by counteracting the luteinizing hormone-induced homologous desensitization via upregulation of the activity of cytochrome P450c17 and luteinizing hormone receptor binding sites [10,11]. Moreover, the excessive insulin synergizes with androgen to prematurely luteinize granulosa cells, leading to follicle maturation arrest and anovulation [12]. Furthermore, proinflammatory cytokines have also been demonstrated to upregulate the activity of cytochrome P450c17 [13]. The goals of therapy for patients with PCOS include improving hyperandrogenic features, managing metabolic abnormalities, and ovulation induction or contraception depending on whether a pregnancy is pursued.

More than a third of women with PCOS suffered from metabolic syndrome [14]. The severity of metabolic syndrome and the phenotype of PCOS are strongly associated with the degree of hyperandrogenism and hyperinsulinemia [15,16]. Decreasing plasma insulin level and improving insulin resistance in patients with PCOS not only benefit hyperandrogenism and ovulation but also reduce cardiovascular risks [1,12,17]. Besides lifestyle modification, metformin is commonly used as an insulin sensitive agent that reduces fasting glucose, improving insulin resistance in patients with PCOS [18]. Combination therapy with metformin and clomiphene also showed positive results for ovulation in patients with PCOS [19]. However, emerging studies on phytomedicine as well as complementary medicine had shown promising results in the treatment of PCOS.

Curcumin, also known as turmeric, is a polyphenol derived from curcumin longa, and is traditionally used in various Asian cuisine [20]. Recently, curcumin has been studied to adjunctly treat broad spectrum of disease from type 2 diabetes mellitus to telogen effluvium [21]. Curcumin elicits antidiabetic effects via several mechanisms, including the increase in glycolysis and glycogen synthesis and the decrease in gluconeogenesis in the liver, as well as the increase in glucose uptake, glycolysis, and glycogen synthesis in the skeletal muscle [22]. Curcumin has also been known to reduce plasma cholesterol and triglyceride by increasing the activity of lipoprotein lipase and through mechanisms which alter lipid and cholesterol gene expression [23,24]. In addition, the anti-inflammatory effects of curcumin have been demonstrated to reduce the oxidative stress in patients with PCOS [25,26]. Previous literature reveals that curcumin significantly improves fasting blood glucose and triglyceride in patients with metabolic syndrome [27]. In vivo study further demonstrates similar effects in the PCOS model [28]. However, the effects of curcumin on metabolic abnormalities in patients with PCOS are not conclusive. The present study aims to evaluate the effects of curcumin on glycemic control and lipid profile in patients with PCOS.

## 2. Materials and Methods

### 2.1. Study Design

The present study is a systematic review and meta-analysis of randomized control trials. The primary aim is to investigate the effects of curcumin on glycemic control in patients with PCOS, which is assessed by fasting glucose, fasting insulin, Homeostasis Model Assessment of Insulin Resistance (HOMA-IR), and quantitative insulin sensitivity check index (QUICKI). The secondary aim is to investigate the effects of curcumin on lipid profile, which is assessed by plasma high-density lipoprotein (HDL), low-density lipoprotein (LDL), triglyceride, and total cholesterol. This study was registered with the International Prospective Register of Systematic Reviews (PROSPERO registration number CRD42021223898) and abides the Preferred Reporting Items for Systematic Review and Meta-Analysis (PRISMA) statement [29].

### 2.2. Search Strategy

Two authors (Y.-J. Chien and C.-Y. Chang) searched five electronic databases from the inception through 18 November 2020, including PubMed, Embase, Scopus, Web of Science, and Cochrane Library. Subject headings (MeSH terms in PubMed and Cochrane Library, and Emtree terms in Embase) and search field tags of title, abstracts and keywords were used to facilitate searching. Terms used for searching relevant records included: “polycystic ovary syndrome”, “polycystic ovarian syndrome”, “Stein-Leventhal syndrome”, “sclerocystic ovarian degeneration”, “sclerocystic ovary syndrome”, “sclerocystic ovary”, “sclerocystic ovaries”, “curcumin”, “curcumins”, “curcuminoid”, “curcuminoids”, “curcuma longa”, “tumeric”, “turmeric”, “curqfen”, “theracurmin”, “nanocurcumin”, “turmeric yellow”, and “diferuloylmethane”. Supplementary Table S1 presents the detailed search strategy. The records identified from the databases were screened by titles, abstracts, and keywords. A full-text review was then carried out on those with potential eligibility. The authors also manually searched the references that were cited in the included studies to retrieve potentially eligible studies.

### 2.3. Eligibility Criteria

Studies were considered eligible if they met the following criteria: (a) the study was a randomized control trial enrolling patients with PCOS; (b) the study compared curcumin with placebo with regard to the outcomes of interest; (c) the study presented information that could be used to calculate the effect estimates for meta-analysis. Studies were not excluded according to publication date, country or language. All studies were selected against the eligibility criteria by two authors (Y.-J. Chien and C.-Y. Chang). Disagreements in the study selection were resolved through discussion and consensus with the third author (M.-Y. Wu)

### 2.4. Risk of Bias Assessment

The revised Cochrane Risk of Bias Tool 2 was used to assess the methodological quality of the included studies [30]. Disagreements were resolved by discussion or consensus with a third reviewer (M.-Y. Wu).

### 2.5. Data Extraction

Data extraction was performed by two reviewers (Y.-J. Chien and C.-Y. Chang) from the included studies. The required information included the author’s name, year of publication, country, number and mean age of patients, dosing regimen and duration of curcumin therapy, diagnostic criteria of PCOS, and the effect estimates of curcumin on the outcomes of interest.

### 2.6. Statistical Analysis

The effects of curcumin on the continuous outcome variables were estimated by comparing the mean difference (MD) and standard deviation of changes before and after the therapy in the curcumin group with those in the placebo group (i.e., curcumin–placebo). The MD and 95% confidence interval (CI) were then calculated for each study using the aforementioned information. Alternatively, the MD and 95% CI were directly extracted if they were reported in the study. The pooled MD was synthesized using the inverse variance method with the random-effects model (DerSimonian–Laird estimator) [31,32]. Statistical heterogeneity was assessed by the Cochran’s Q statistic and quantified by the I^2^ statistic. In addition, an a priori meta-regression was planned to explore the influence of daily dose of curcumin and duration of therapy on the pooled effect estimates that are pooled from ≥10 studies [33].

In order to evaluate whether the results of the conventional meta-analysis were subject to type I or type II error due to sparse data and lack of power, trial sequential analysis (TSA) was applied to calculate the diversity-adjusted required information size (RIS) and trial sequential monitoring boundaries [34]. The models were set at an alpha of 5% and a power of 80% for all outcomes. Influence analysis was carried out as a sensitivity analysis by omitting one study at a time and recalculating the pooled results from each subset of the studies. Finally, if the pooled results were synthesized from greater than 10 studies, a contour-enhanced funnel plot and Egger’s test were conducted to evaluate whether the publication bias existed [33,35,36]. In the case of significant asymmetry indicated by Egger’s test, the trim-and-fill method was performed to identify the missing studies [37]. The statistical analyses were carried out using R software version 3.6.1 (R Foundation for Statistical Computing, Vienna, Austria) [38] with “dmetar”, “meta”, and “metafor” packages. TSA was performed with TSA software version 0.9.5.10 Beta (Copenhagen Trial Unit, Copenhagen, Denmark) [39]. A *p*-value < 0.05 was considered statistically significant.

## 3. Results

### 3.1. Study Identification and Selection

**Figure 1:**
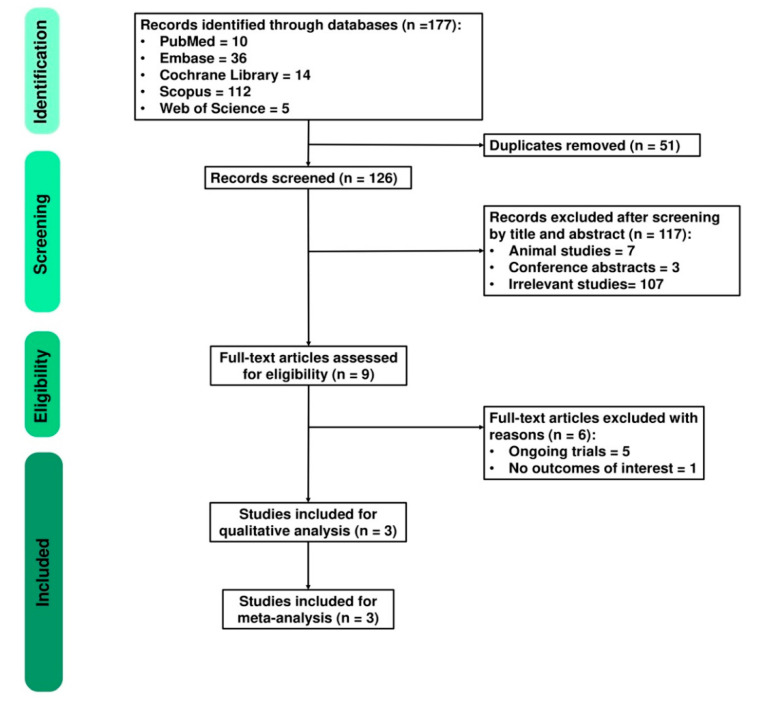
PRISMA flow diagram.

A total of 177 studies were identified from five databases, including PubMed (*n* = 10), EMBASE (*n* = 36), Cochrane Library (*n* = 14), SCOPUS (*n* = 112), and Web of Science (*n* = 5). After removing 51 duplicates, the remaining studies were screened for eligibility. A total of 117 records were excluded due to irrelevant topics by screening titles and abstracts. Therefore, nine studies were assessed for full-text review. Six studies were excluded due to ongoing trials and not having outcomes of interest. Finally, three studies involving 168 patients were included. The detailed PRISMA flow diagram is presented in Figure 1.

### 3.2. Study Characteristics and Risk of Bias Assessment

The characteristics of the included studies are presented in Table 1. All studies are randomized control trials. The dosing regimen of curcumin ranges from 500 mg once per day [40] to 500 mg three times per day [41]. Maltodextrin is used as placebo in one study [41], starch is used in another [40], and the other does not specify what consists of placebo [42]. The duration of therapy ranges from 6 weeks [42] to 12 weeks [40,41]. The diagnosis of PCOS is based on Rotterdam criteria in all the included studies [3]. In addition, the datasets extracted from each included study with regard to the outcomes of interest are presented in Table 2. Moreover, all the included studies are of low risk of overall bias despite some concerns raised from the randomization process, measurement of the outcome, and deviation from intended interventions. Notably, despite the measures taken to ensure the patient’s adherence (e.g., returning the medication containers, and the reminders of taking medication by cell phone), deviation from intended interventions may, though unlikely, have occurred. The detailed risk of bias assessment for each included study is presented in Supplementary Figure S1.

### 3.3. Outcomes

#### 3.3.1. Glycemic Control

**Figure 2:**
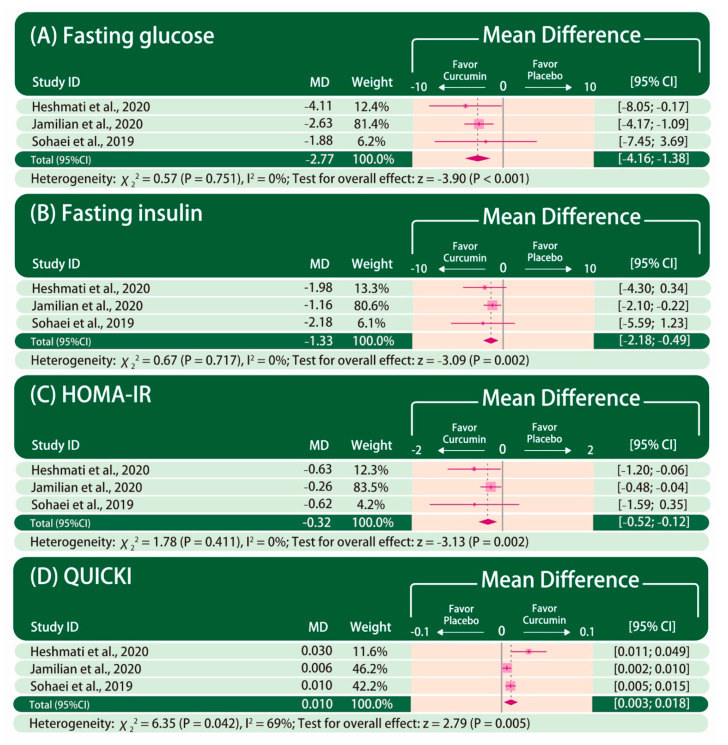
Forest plot of glycemic control. The mean difference between curcumin and placebo is calculated as the value in the curcumin group subtracted by that in the placebo group (curcumin–placebo). CI: confidence interval. MD: mean difference.

The forest plot of glycemic control is presented in Figure 2. Fasting glucose, fasting insulin, HOMA-IR, and QUICKI were reported in all the included studies. The improvement in fasting glucose (MD: −2.77, 95% CI: −4.16 to −1.38; *p* < 0.001; I^2^ = 0%), fasting insulin (MD: −1.33, 95% CI: −2.18 to −0.49; P = 0.002; I^2^ = 0%), HOMA-IR (MD: −0.32, 95% CI: −0.52 to −0.12; P = 0.002; I^2^ = 0%), and QUICKI (MD: 0.010, 95% CI: 0.003–0.018; P = 0.005; I^2^ = 69%) are significantly greater in patients taking curcumin than those taking placebo.

#### 3.3.2. Lipid Profile

**Figure 3:**
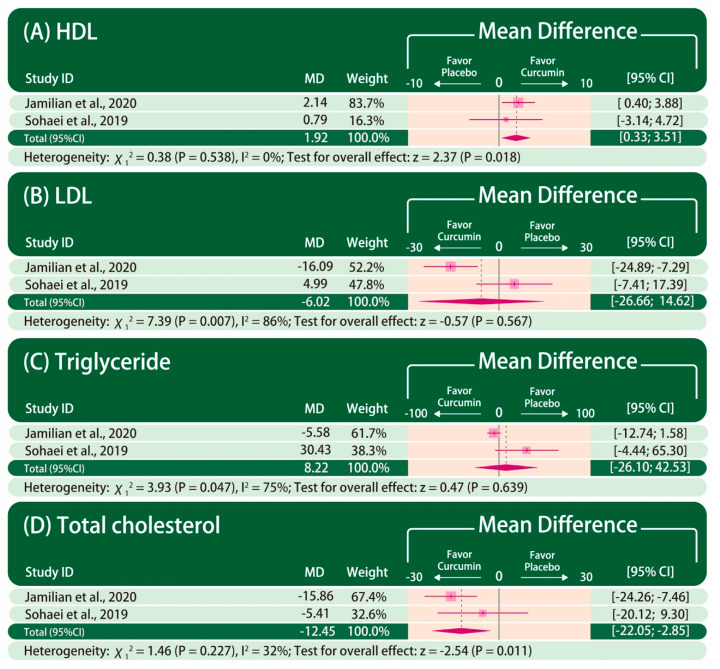
Forest plot of lipid profile. The mean difference between curcumin and placebo is calculated as the value in the curcumin group subtracted by that in the placebo group (curcumin–placebo). CI: confidence interval. MD: mean difference.

The forest plot of the lipid profile is presented in Figure 3. HDL, LDL, triglyceride, and total cholesterol were reported in only two studies [40,42]. The improvement in HDL (MD: 1.92, 95% CI: 0.33–3.51; P = 0.018; I^2^ = 0%) and total cholesterol (MD: −12.45, 95% CI: −22.05 to −2.85; P = 0.011; I^2^ = 32%) are significantly greater in patients taking curcumin than those taking placebo. In contrast, there is no statistically significant difference in the improvement in LDL (MD: −6.02, 95% CI: −26.66 to 14.62; P = 0.567; I^2^ = 86%) and triglyceride (MD: 8.22, 95% CI: −26.10 to 42.53; P = 0.639; I^2^ = 75%) between patients taking curcumin and those taking placebo.

#### 3.3.3. Influence Analysis

The influence analysis is presented in Supplementary Figure S2. For the fasting glucose, fasting insulin and QUICKI, the influence analysis reveals that the pooled point estimates after omitting each included study one at a time lie within the 95% CI of the overall pooled results for these outcomes. In contrast, the influence analysis of HOMA-IR reveals that the pooled point estimate after omitting the study by Jamilian et al. lies outside of the 95% CI of the overall pooled result. The influence analysis of the lipid profile was not performed due to limited number of studies in each outcome.

#### 3.3.4. Trial Sequential Analysis

The cumulative Z-curve has reached the estimated RIS and has passed the traditional significance boundary in favor of curcumin in the TSA of fasting glucose, fasting insulin, HOMA-IR, and QUICKI (Supplementary Figures S3–S6). In the TSA of total cholesterol, although the cumulative Z-curve has not yet reached the estimated RIS, it passes the adjusted significance boundary in favor of curcumin (Supplementary Figure S7). In contrast, in the TSA of HDL, although the cumulative Z-curve passes the traditional significance boundary, it has not yet reached the estimated RIS, and has not passed the adjusted significance boundary in favor of curcumin (Supplementary Figure S8). In the TSA of LDL and triglyceride, the cumulative Z-curves have not reached the estimated RIS (2432 for LDL and 3609 for triglyceride), and the sequential monitoring boundaries are ignored due to too few patients relative to the estimated RIS (Supplementary Figures S9 and S10).

#### 3.3.5. Meta-Regression and Publication Bias

Despite the pre-planned attempts to evaluate the effects of daily dose of curcumin and duration of therapy on the pooled effect estimates, a meta-regression is not performed due to limited number of studies eligible for inclusion. Similarly, publication bias is not assessed by a funnel plot and performing Egger’s test due to few numbers of included studies.

## 4. Discussion

**Figure 4:**
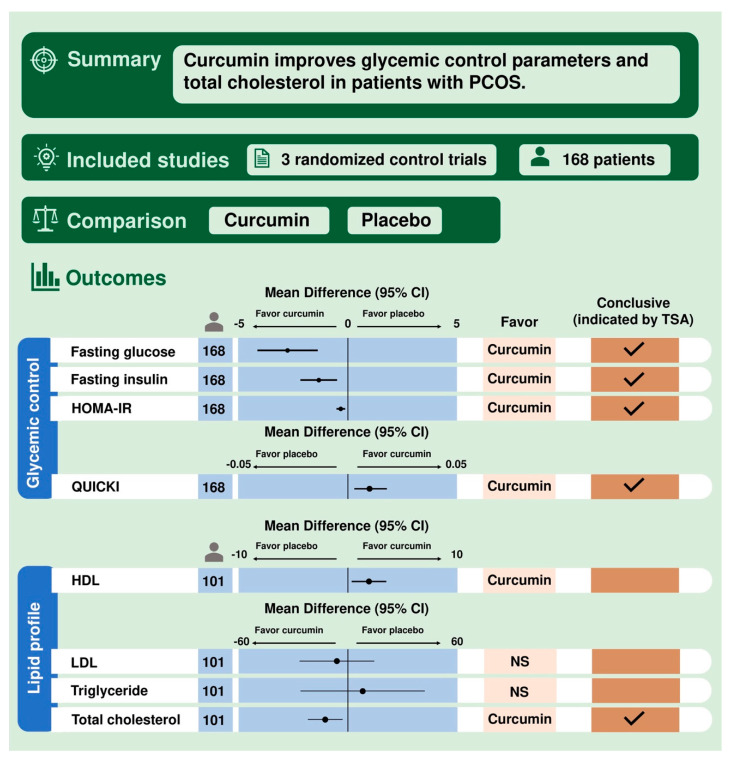
Visual summary abstract. CI: confidence interval. HDL: high-density lipoprotein. HOMA-IR: Homeostasis Model Assessment of Insulin Resistance. LDL: low-density lipoprotein. NS: non-significant. PCOS: polycystic ovary syndrome. QUICKI: quantitative insulin sensitivity check index. TSA: trial sequential analysis.

The principal finding of the present study is that patients with PCOS taking curcumin have significantly greater improvement in glycemic control than those taking placebo, reflected by the fasting glucose, fasting insulin, HOMA-IR and QUICKI. In addition, curcumin also shows beneficial effects in improving lipid profile, including HDL and total cholesterol. In contrast, there is no statistically significant difference in LDL and triglyceride between patients taking curcumin and placebo. The TSA shows that the results of the current meta-analysis with regard to the fasting glucose, fasting insulin, HOMA-IR, QUICKI, and total cholesterol are conclusive. In contrast, the TSA indicates that the effects of curcumin on HDL, LDL, and triglyceride are not yet conclusive, and thus more large-scaled trials to determine these results are required. A visual summary abstract is presented in Figure 4.

Among the included studies, the curcumin dosage ranged from 500 mg to 1500 mg per day and the treatment period ranged from 6 weeks to 12 weeks. All of the patients were diagnosed with Rotterdam criteria and were studied in Iran. One study by Sohaei et al. mentioned the concentration and detailed manufacturing of curcumin with 95% of standardized turmeric powder. One study took place in infertility center but none of the included studies discuss the effects of curcumin in ovulation or reproduction.

The effects of curcumin on glycemic control and lipid metabolism are complex and involve several mechanisms that underlie the observation in the present study. Curcumin stimulates insulin-mediated glucose uptake by the phosphatidylinositol 3-kinase (PI3K)/Akt pathway, which in turn upregulates the translocation of glucose transporter 4 (GLUT4) to the membrane of adipocyte and skeletal muscle, leading to an increase in glucose uptake [43,44]. Curcumin also activates adenosine monophosphate-activated protein kinase, which not only suppresses gluconeogenesis in hepatocyte via inhibiting glucose-6-phosphatase and phosphoenolpyruvate carboxykinase [45,46], but also enhances GLUT4 translocation and glucose uptake in adipocytes [47]. Moreover, curcumin improves glucose homeostasis by activating glucose transporter 2 and glucokinases in liver via increasing the transcription of peroxisome proliferator-activated receptor-gamma (PPAR-γ) [48,49]. In the study by Jamilian et al., significant upregulation of PPAR-γ has been observed after taking curcumin for 12 weeks [40]. With regard to lipid metabolism, curcumin upregulates LDL receptors and inhibits the synthesis of cholesterol and triglyceride in hepatocyte [50,51]. It also promotes cholesterol catabolism and fecal excretion of bile acids [52].

The anti-inflammatory property of curcumin may also play a role in glucose and lipid metabolisms and may mitigate hyperandrogenism. Proinflammatory cytokines, such as tumor necrosis factor-α (TNF-α), are found to be significantly higher in patients with PCOS [53]. TNF-α has been known to stimulate serine phosphorylation of insulin receptor substrate 1, resulting in insulin resistance [54]. Moreover, serine phosphorylation of cytochrome P450c17 enhances the activity of 17,20-lyase and promotes thecal production of androgen [55]. Curcumin inhibits PI3K/Akt/mechanistic target of rapamycin (mTOR) signaling pathway, resulting in the degradation of nuclear factor kappa-light-chain-enhancer of activated B cells (NF-κB) and downregulation of TNF-α and other proinflammatory cytokines [56,57]. Curcumin has been demonstrated to significantly reduce the plasma levels of TNF-α and interleukin (IL)-6 [58,59] and, subsequently, improves insulin sensitivity and decreases obesity-induced insulin resistance [60,61]. In addition, patients with PCOS are associated with higher oxidative stress [62]. A recent study has demonstrated beneficial effects of curcumin on upregulating the gene expression of peroxisome proliferator-activated receptor-gamma coactivator 1 alpha (PGC-1α), which in turn increases the activity of glutathione peroxidase, reducing the oxidative stress expression [25].

Curcumin may have promising effects in improving hyperandrogenism, though this is not demonstrated in the present study because meta-analysis, which evaluates and reports the influence of curcumin on thecal androgen production, is not performed due to the limited number of studies. In the study by Heshmati et al., the plasma levels of dehydroepiandrosterone (DHEA) are significantly reduced in patients taking curcumin compared to those taking placebo for 12 weeks [41]. This observation is likely attributed to the downregulation of the activity of cytochrome P450c17. Curcumin has been demonstrated to reduce the activity of cytochrome P450c17 in a dose-dependent manner in vitro [63]. In an animal model, the expression of cytochrome P450c17 in the ovaries of the curcumin-treated mice is reduced to the same level as in those of the wild-type ones [64]. Together with the anti-inflammatory property of curcumin, these results collectively support the notion that curcumin inhibits the activity of cytochrome P450c17, and thereby reduces the synthesis of DHEA.

There are several limitations of the present study. First, the number of studies that are eligible for inclusion is limited. Nevertheless, a TSA is performed to evaluate the robustness of the results. While some of the outcomes remain inconclusive and require more investigation, most of the results in the present study are conclusive as indicated by the TSA. However, all of the included studies were from Iran. The results of our meta-analysis should be carefully interpreted with populations other than Middle Eastern ethnicity. Second, the dosing regimen and duration of curcumin therapy varied across studies. The daily dosage of curcumin in the included studies ranged from 500 mg to 1500 mg. However, the duration of curcumin was relatively short from 6 weeks up to 12 weeks. Despite the pre-planned meta-regression to assess the effects of these covariates on the pooled effect estimates, it was not performed due to limited number of studies. Third, the detailed information regarding the preparation of the capsule and concentration of curcumin is not mentioned in the included studies. It is well documented that curcumin has poor bio-availability due to low absorption in intestines [65]. The difference in capsule design may affect the plasma concentration and the duration of the effects of curcumin. Finally, the effects of curcumin on sex hormone abnormalities in patients with PCOS are not assessed in the present study due to a lack of investigation in the included studies. Although the comprehensive therapy for PCOS is beyond the scope of the present study, the potential of curcumin in lowering plasma levels of androgen, as demonstrated in the study by Heshmati et al., is promising. Further investigation to confirm this result is advocated.

## 5. Conclusions

Curcumin may improve glycemic control and lipid metabolism in patients with PCOS and metabolic abnormality without significant adverse effects. Further studies are advocated to investigate the potential effects of curcumin on hyperandrogenism.

## Figures and Tables

**Table 1:** Study characteristics.

Scheme	Country	Study Design	Duration (Weeks)	Sample Size		Mean Age ^†^		Regimen		Diagnostic Criteria
				Intervention	Control	Intervention	Control	Intervention	Control	
Sohaei et al. 2019 [42]	Iran	Double-blind RCT	6	27	24	29.4 (5.3)	29.6 (5.0)	Curcumin 500 mg BID	Placebo BID	Rotterdam criteria
Heshmati et al. 2020 [41]	Iran	Double-blind RCT	12	34	33	31.0 (5.2)	30.8 (8.0)	Curcumin 500 mg TID	Placebo (maltodextrin) TID	Rotterdam criteria
Jamilian et al. 2020 [40]	Iran	Double-blind RCT	12	26	24	28.6 (4.7)	27.2 (3.4)	Curcumin 500 mg QD	Placebo (starch) QD	Rotterdam criteria

^†^ Mean age is presented as mean (standard deviation). BID: twice per day; QD: once per day; RCT: randomized control trial; TID: three times per day.

**Table 2:** Measurement of glycemic control and lipid profile in the curcumin and placebo group.

Study	Mean Difference (Standard Deviation) ^†^		Mean Difference (95% Confidence Interval) ^‡^
	Curcumin	Placebo	Curcumin−Placebo
**Fasting glucose (mg/dL)**			
Sohaei et al. 2019 [42]	2.62 (9.48)	4.50 (10.80)	-
Heshmati et al. 2020 [41]	−5.09 (7.29)	−0.98 (9.11)	-
Jamilian et al. 2020 [40]	-	-	−2.63 (−4.21, −1.05)
**Fasting insulin (μIU/mL)**			
Sohaei et al. 2019 [42]	−3.06 (6.44)	−0.88 (5.93)	-
Heshmati et al. 2020 [41]	−1.35 (4.90)	0.63 (4.77)	-
Jamilian et al. 2020 [40]	-	-	−1.16 (−2.12, −0.19)
**HOMA-IR**			
Sohaei et al. 2019 [42]	−0.69 (1.87)	−0.07 (1.65)	-
Heshmati et al. 2020 [41]	−0.47 (1.22)	0.16 (1.17)	-
Jamilian et al. 2020 [40]	-	-	−0.26 (−0.48, −0.03)
**QUICKI**			
Sohaei et al. 2019 [42]	0.010 (0.010)	0.000 (0.010)	-
Heshmati et al. 2020 [41]	0.020 (0.040)	−0.010 (0.040)	-
Jamilian et al. 2020 [40]	-	-	0.006 (0.001, 0.010)
**HDL (mg/dL)**			
Sohaei et al. 2019 [42]	1.82 (6.30)	1.03 (7.99)	-
Jamilian et al. 2020 [40]	-	-	2.14 (0.36, 3.92)
**LDL (mg/dL)**			
Sohaei et al. 2019 [42]	3.20 (21.82)	−1.79 (23.34)	-
Jamilian et al. 2020 [40]	-	-	−16.09 (−25.11, −7.06)
**Triglyceride (mg/dL)**			
Sohaei et al. 2019 [42]	8.81 (70.73)	−21.62 (53.96)	-
Jamilian et al. 2020 [40]	-	-	−5.58 (−12.93, 1.77)
**Total cholesterol (mg/dL)**			
Sohaei et al. 2019 [42]	−3.33 (18.58)	2.08 (33.67)	-
Jamilian et al. 2020 [40]	-	-	−15.86 (−24.48, −7.24)

^†^ The mean difference refers to the changes before and after the therapy in the curcumin and placebo group. ^‡^ The mean difference refers to the difference in changes before and after the therapy between the curcumin and placebo group. HDL: high-density lipoprotein; HOMA-IR: Homeostasis Model Assessment of Insulin Resistance; LDL: low-density lipoprotein; QUICKI: quantitative insulin sensitivity check index.

## Supplementary Materials

The following are available online at https://www.mdpi.com/2072-6643/13/2/684/s1, Figure S1: Risk of bias summary and graph, Figure S2: Influence analysis of fasting glucose, fasting insulin, Homeostasis Model Assessment of Insulin Resistance (HOMA-IR) and quantitative insulin sensitivity check index (QUICKI), Figure S3: Trial sequential analysis of fasting glucose, Figure S4: Trial sequential analysis of fasting insulin, Figure S5: Trial sequential analysis of HOMA-IR, Figure S6: Trial sequential analysis of QUICKI, Figure S7: Trial sequential analysis of total cholesterol, Figure S8: Trial sequential analysis of HDL, Figure S9: Trial sequential analysis of LDL, Figure S10: Trial sequential analysis of triglyceride, Table S1: Detailed search strategy.
