# Supplementary material for: Effects of Curcumin on Glycemic Control and Lipid Profile in Polycystic Ovary Syndrome: Systematic Review with Meta-Analysis and Trial Sequential Analysis
Source: Nutrients. 2021 Feb 21;13(2):684. doi: 10.3390/nu13020684 (PMC7924860; doi:10.3390/nu13020684)
Supplement: Supplementary file 1 [file nutrients-13-00684-s001.pdf]

Supplementary materials

Effects of curcumin on glycemic control and lipid profile in polycystic ovary syndrome: Systematic review with meta-analysis and trial sequential analysis

Yung-Jiun Chien, Chun-Yu Chang, Meng-Yu Wu, Chih-Hao Chen, Yi-Shiung Horng, Hsin-Chi Wu

Supplementary Table S1. Detailed search strategy

Database	Search strategy
PubMed	("Curcumin"[MeSH Terms] OR "curcumin/administration and dosage"[MeSH Terms] OR "curcumin/analogs and derivatives"[MeSH Terms] OR "curcumin/pharmacology"[MeSH Terms] OR "curcumin/therapeutic use"[MeSH Terms] OR ("Curcumin"[Title/Abstract] OR "curcumins"[Title/Abstract] OR "curcuminoid"[Title/Abstract] OR "curcuminoids"[Title/Abstract] OR "curcuma longa"[Title/Abstract] OR "tumeric"[Title/Abstract] OR "turmeric"[Title/Abstract] OR "curqfen"[Title/Abstract] OR "theracurmin"[Title/Abstract] OR "nanocurcumin"[Title/Abstract] OR "turmeric yellow"[Title/Abstract] OR "diferuloylmethane"[Title/Abstract]) OR ("Curcumin"[All Fields] OR "curcumins"[All Fields] OR "curcuminoid"[All Fields] OR "curcuminoids"[All Fields] OR "curcuma longa"[All Fields] OR "tumeric"[All Fields] OR "turmeric"[All Fields] OR "curqfen"[All Fields] OR "theracurmin"[All Fields] OR "nanocurcumin"[All Fields] OR "turmeric yellow"[All Fields] OR "diferuloylmethane"[All Fields])) AND ("polycystic ovary syndrome"[MeSH Terms] OR "polycystic ovary syndrome/diet therapy"[MeSH Terms] OR "polycystic ovary syndrome/drug therapy"[MeSH Terms] OR "polycystic ovary syndrome/prevention and control"[MeSH Terms] OR ("ovary syndrome polycystic"[Title/Abstract] OR "syndrome polycystic ovary"[Title/Abstract] OR "stein leventhal syndrome"[Title/Abstract] OR "stein leventhal syndrome"[Title/Abstract] OR "Sclerocystic Ovarian Degeneration"[Title/Abstract] OR "Sclerocystic Ovary Syndrome"[Title/Abstract] OR "Polycystic Ovarian Syndrome"[Title/Abstract] OR "ovarian syndrome polycystic"[Title/Abstract] OR "Polycystic Ovary Syndrome 1"[Title/Abstract] OR "Sclerocystic Ovaries"[Title/Abstract] OR "Sclerocystic Ovary"[Title/Abstract]) OR ("ovary syndrome polycystic"[All Fields] OR "syndrome polycystic ovary"[All Fields] OR "stein leventhal syndrome"[All Fields] OR "stein leventhal syndrome"[All Fields] OR "syndrome stein leventhal"[All Fields] OR "Sclerocystic Ovarian Degeneration"[All Fields] OR ("polycystic ovary syndrome"[MeSH Terms] OR ("polycystic"[All Fields] AND "ovary"[All Fields] AND "syndrome"[All Fields]) OR "polycystic

	ovary syndrome"[All Fields] OR ("ovarian"[All Fields] AND "degeneration"[All Fields] AND "sclerocystic"[All Fields])) OR "Sclerocystic Ovary Syndrome"[All Fields] OR "Polycystic Ovarian Syndrome"[All Fields] OR "ovarian syndrome polycystic"[All Fields] OR "Polycystic Ovary Syndrome 1"[All Fields] OR "Sclerocystic Ovaries"[All Fields] OR ("polycystic ovary syndrome"[MeSH Terms] OR ("polycystic"[All Fields] AND "ovary"[All Fields] AND "syndrome"[All Fields]) OR "polycystic ovary syndrome"[All Fields] OR ("ovary"[All Fields] AND "sclerocystic"[All Fields])) OR "Sclerocystic Ovary"[All Fields] OR "polycystic ovary syndrome"[All Fields] OR "polycystic ovarian disease"[All Fields] OR "ovary polycystic disease"[All Fields]))
Embase	('ovary syndrome, polycystic' OR 'or syndrome, polycystic ovary' OR 'stein-leventhal syndrome' OR 'syndrome, stein-leventhal' OR 'sclerocystic ovarian degeneration' OR 'ovarian degeneration, sclerocystic' OR 'sclerocystic ovary syndrome' OR 'polycystic ovarian syndrome' OR 'ovarian syndrome, polycystic' OR 'polycystic ovary syndrome 1' OR 'sclerocystic ovaries' OR 'ovary, sclerocystic' OR 'sclerocystic ovary' OR 'ovary polycystic disease'/exp OR 'stein leventhal syndrome' OR 'cystic ovary' OR 'micropolycystic ovary' OR 'multiple follicle cyst' OR 'ovary polycystic disease' OR 'ovary polycystic syndrome' OR 'ovary, micropolycystic' OR 'ovary, polycystic' OR 'polycystic ovarian disease' OR 'polycystic ovary' OR 'polycystic ovary disease' OR 'polycystic ovary syndrome' OR 'stein cohen leventhal syndrome' OR 'stein leventhal disease' OR 'syndrome stein leventhal') AND ('curcumin'/exp OR curcumins OR 'curcuminoid'/exp OR curcuminoids OR 'curcuma longa'/exp OR tumeric OR 'turmeric'/exp OR curqfen OR 'theracurmin'/exp OR 'tumeric yellow' OR diferuloylmethane)
Scopus	TITLE-ABS-KEY(("ovary syndrome, polycystic" OR "Syndrome, Polycystic Ovary" OR "Stein-Leventhal Syndrome" OR "Stein Leventhal Syndrome" OR "Syndrome, Stein-Leventhal" OR "Sclerocystic Ovarian Degeneration" OR "Ovarian Degeneration, Sclerocystic" OR "Sclerocystic Ovary Syndrome" OR "Polycystic Ovarian Syndrome" OR "Ovarian Syndrome, Polycystic" OR "Polycystic Ovary Syndrome 1" OR "Sclerocystic Ovaries" OR "Ovary, Sclerocystic" OR "Sclerocystic Ovary" OR "Polycystic ovary syndrome" OR "polycystic ovarian disease" OR "ovary polycystic

	disease") AND ("curcumin" OR "curcumins" OR "curcuminoid" OR "curcuminoids" OR "curcuma longa" OR "tumeric" OR "turmeric" OR "curqfen" OR "theracurmin" OR "nanocurcumin" OR "turmeric yellow" OR "diferuloylmethane"))
Web of Science	TS=(("ovary syndrome, polycystic" OR "Syndrome, Polycystic Ovary" OR "Stein-Leventhal Syndrome" OR "Stein Leventhal Syndrome" OR "Syndrome, Stein-Leventhal" OR "Sclerocystic Ovarian Degeneration" OR "Ovarian Degeneration, Sclerocystic" OR "Sclerocystic Ovary Syndrome" OR "Polycystic Ovarian Syndrome" OR "Ovarian Syndrome, Polycystic" OR "Polycystic Ovary Syndrome 1" OR "Sclerocystic Ovaries" OR "Ovary, Sclerocystic" OR "Sclerocystic Ovary" OR "Polycystic ovary syndrome" OR “polycystic ovarian disease” OR “ovary polycystic disease”) AND ("curcumin" OR "curcumins" OR "curcuminoid" OR "curcuminoids" OR "curcuma longa" OR "tumeric" OR "turmeric" OR "curqfen" OR "theracurmin" OR "nanocurcumin" OR "turmeric yellow" OR "diferuloylmethane"))
Cochrane Library	Search Name:<br>Date Run: 28/11/2020 23:32:33<br>Comment:<br><br>ID Search Hits<br>#1 MeSH descriptor: [Polycystic Ovary Syndrome] explode all trees 1520<br>#2 MeSH descriptor: [Polycystic Ovary Syndrome] explode all trees and with qualifier(s): [diet therapy - DH, drug therapy - DT, prevention & control - PC] 665<br>#3 "ovary syndrome, polycystic" OR "Syndrome, Polycystic Ovary" OR "Stein-Leventhal Syndrome" OR "Stein Leventhal Syndrome" OR "Syndrome, Stein-Leventhal" OR "Sclerocystic Ovarian Degeneration" OR "Ovarian Degeneration, Sclerocystic" OR "Sclerocystic Ovary Syndrome" OR "Polycystic Ovarian Syndrome" OR "Ovarian Syndrome, Polycystic" OR "Polycystic Ovary Syndrome 1" OR "Sclerocystic Ovaries" OR "Ovary, Sclerocystic" OR "Sclerocystic Ovary" OR "Polycystic ovary syndrome" OR “polycystic ovarian disease” OR “ovary polycystic disease”3819<br>#4 {OR #1-#3} 3819<br>#5 MeSH descriptor: [Curcumin] explode all trees and with

	qualifier(s): [administration & dosage - AD, analogs & derivatives - AA, pharmacology - PD, therapeutic use - TU] 213<br>#6 MeSH descriptor: [Curcumin] explode all trees 383<br>#7 MeSH descriptor: [Diarylheptanoids] explode all trees 387<br>#8 MeSH descriptor: [Diarylheptanoids] explode all trees and with qualifier(s): [administration & dosage - AD, analogs & derivatives - AA, pharmacology - PD, therapeutic use - TU] 217<br>#9 "curcumin" OR "curcumins" OR "curcuminoid" OR "curcuminoids" OR "curcuma longa" OR "tumeric" OR "turmeric" OR "curqfen" OR "theracurmin" OR "nanocurcumin" OR "turmeric yellow" OR "diferuloylmethane" 1546<br>#10 {OR #5-#9} 1546<br>#11 #4 AND #10 14

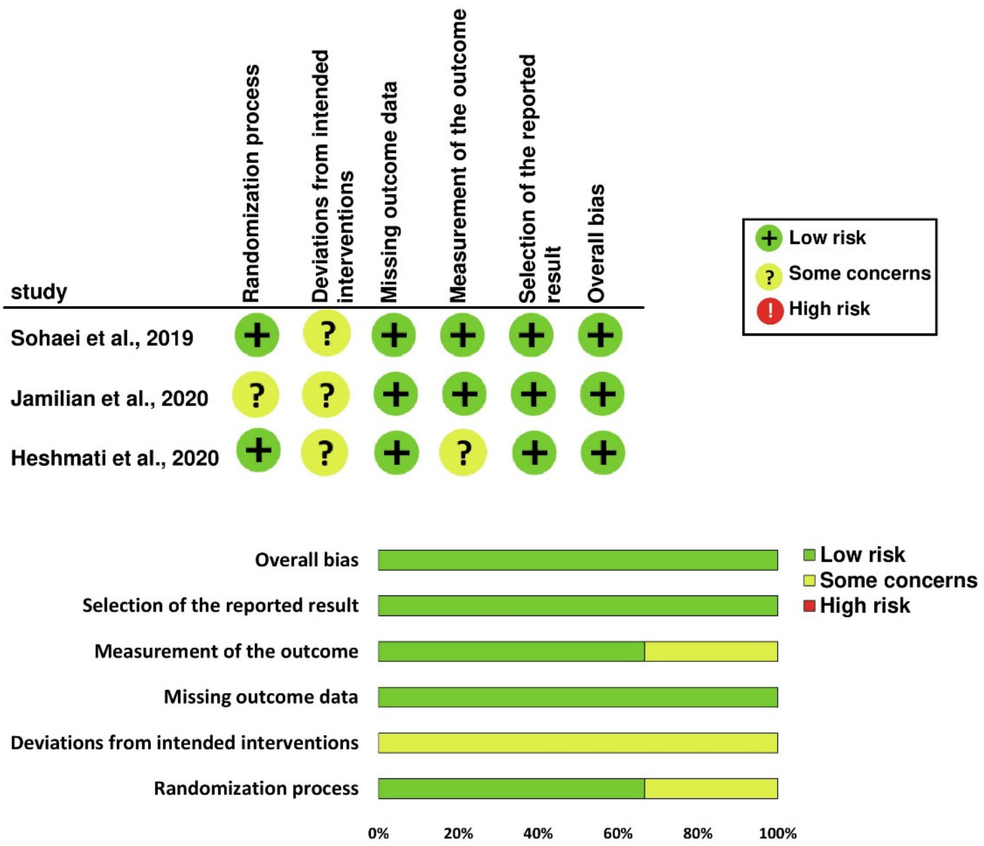

Supplementary Figure S1. Risk of bias summary and graph.

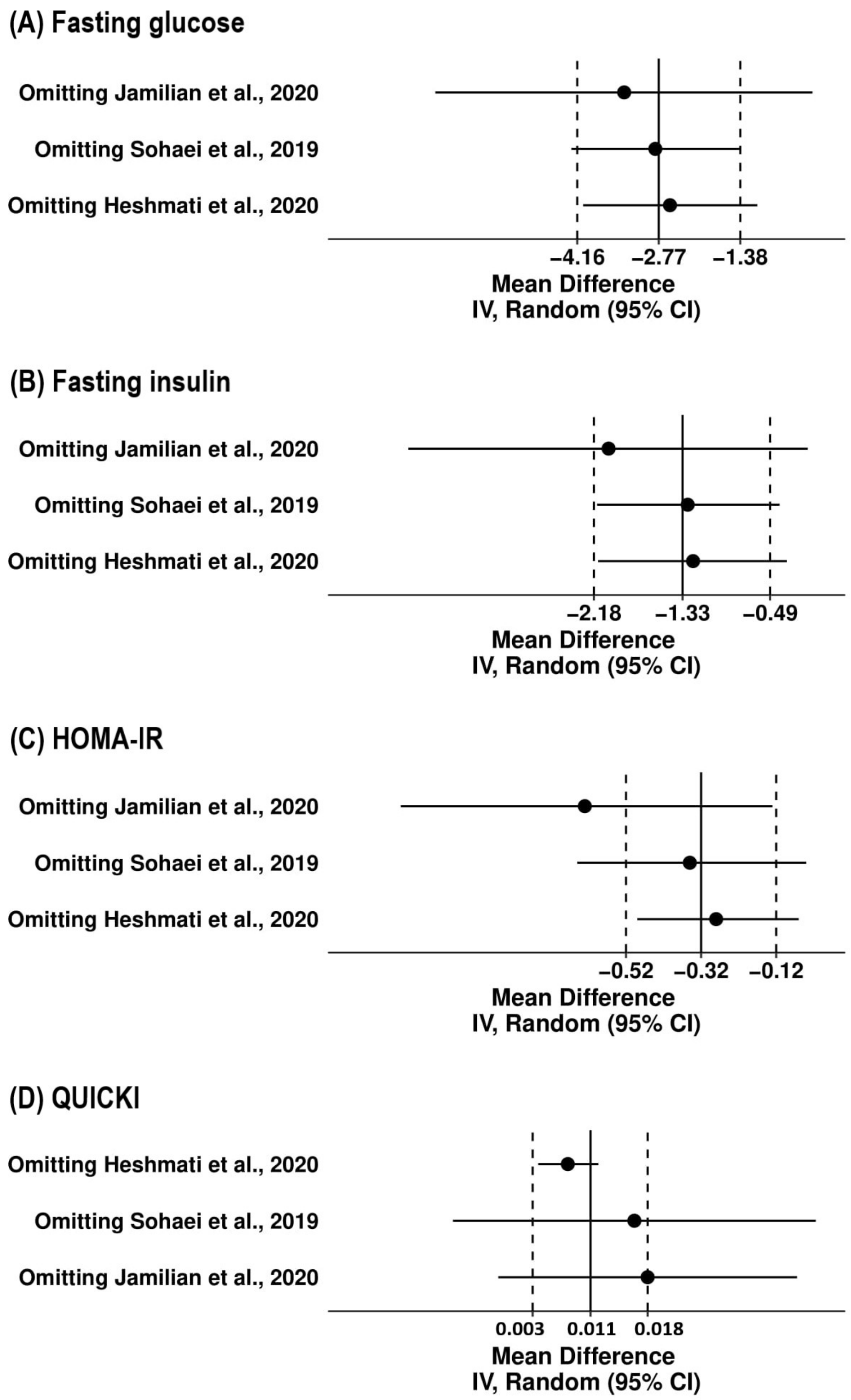

Supplementary Figure S2. Influence analysis of fasting glucose, fasting insulin, Homeostasis Model Assessment of Insulin Resistance (HOMA-IR) and quantitative insulin sensitivity check index (QUICKI). CI: confidence interval. IV: inverse-variance method.

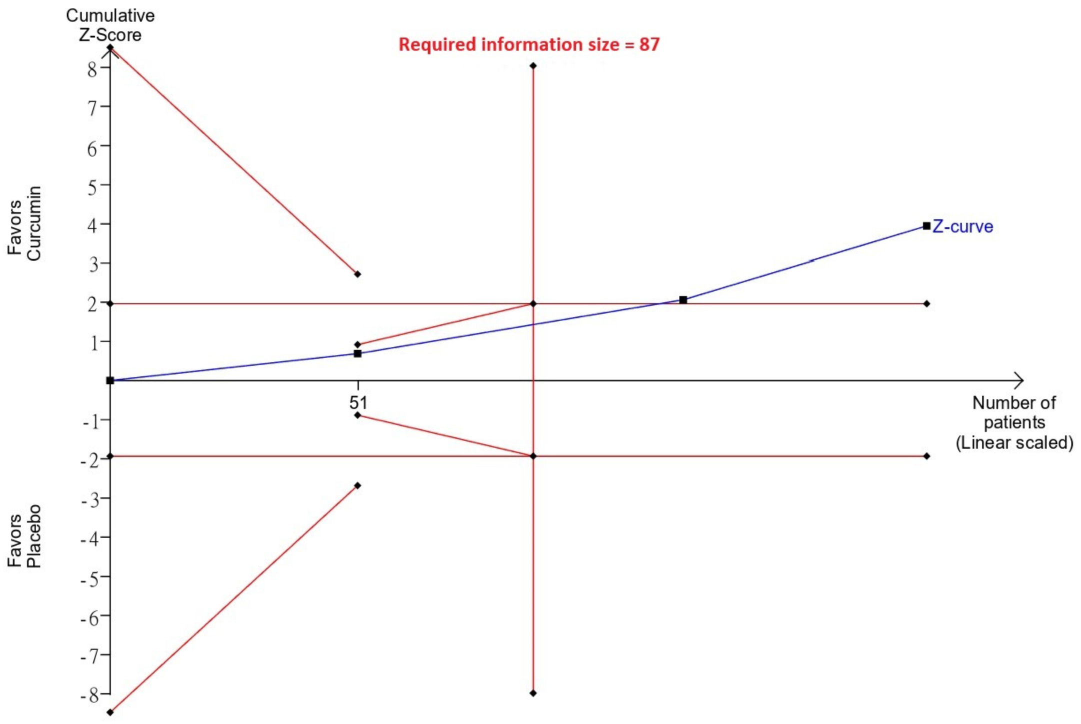

Supplementary Figure S3. Trial sequential analysis of fasting glucose.

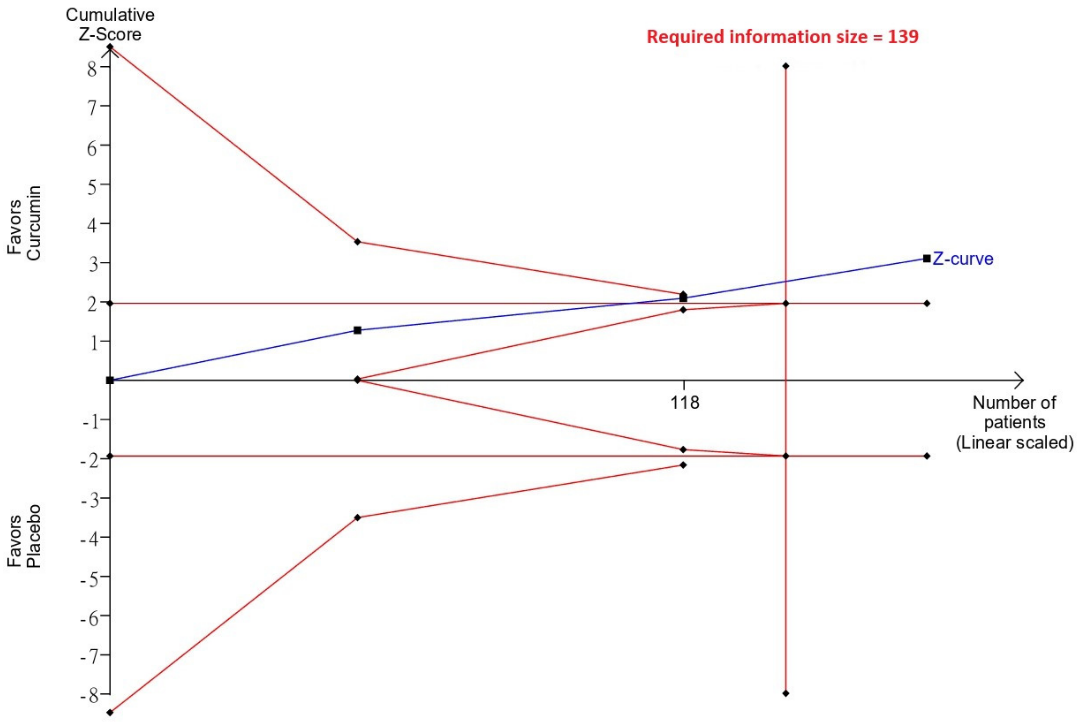

Supplementary Figure S4. Trial sequential analysis of fasting insulin.

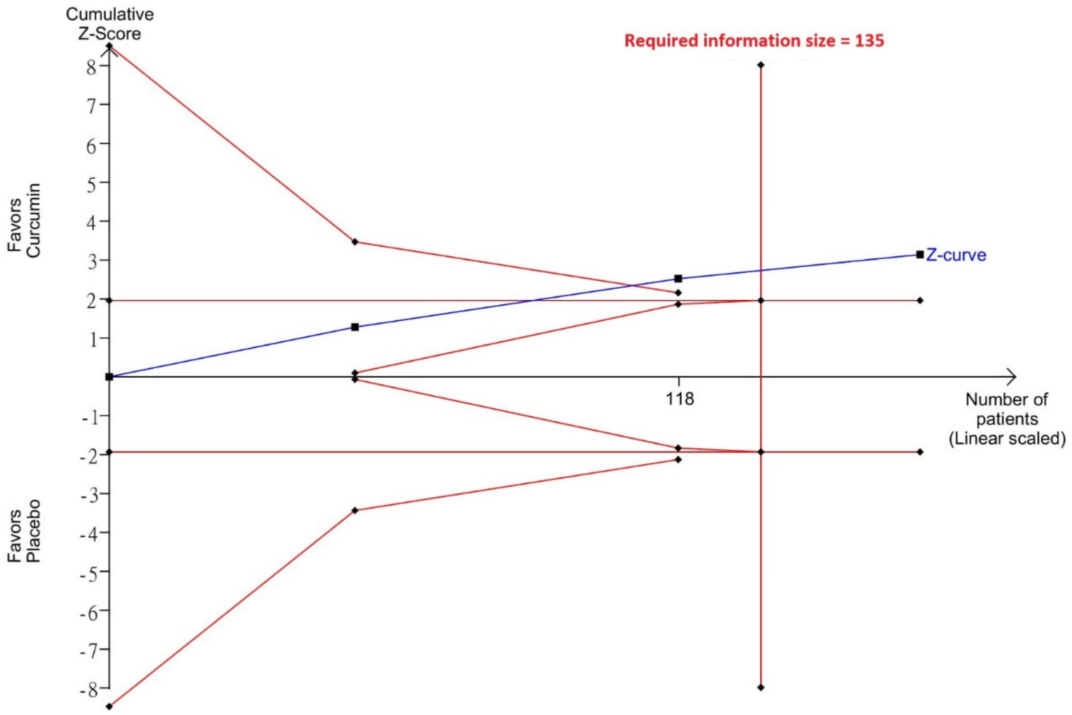

Supplementary Figure S5. Trial sequential analysis of HOMA-IR.

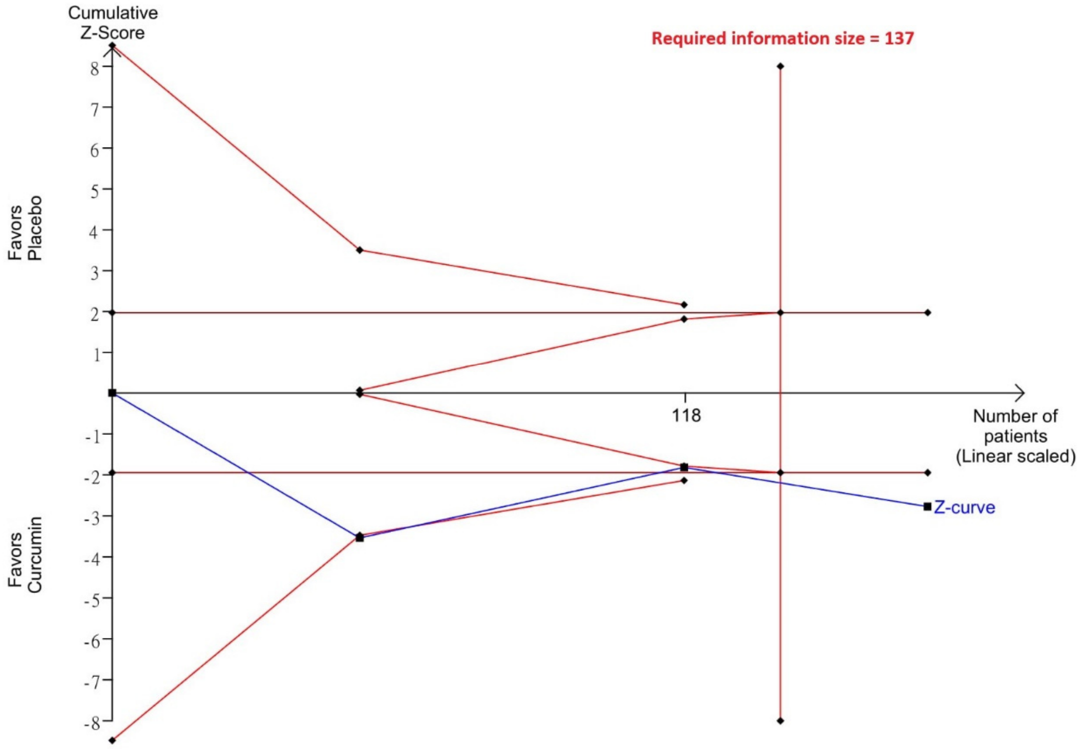

Supplementary Figure S6. Trial sequential analysis of QUICKI.

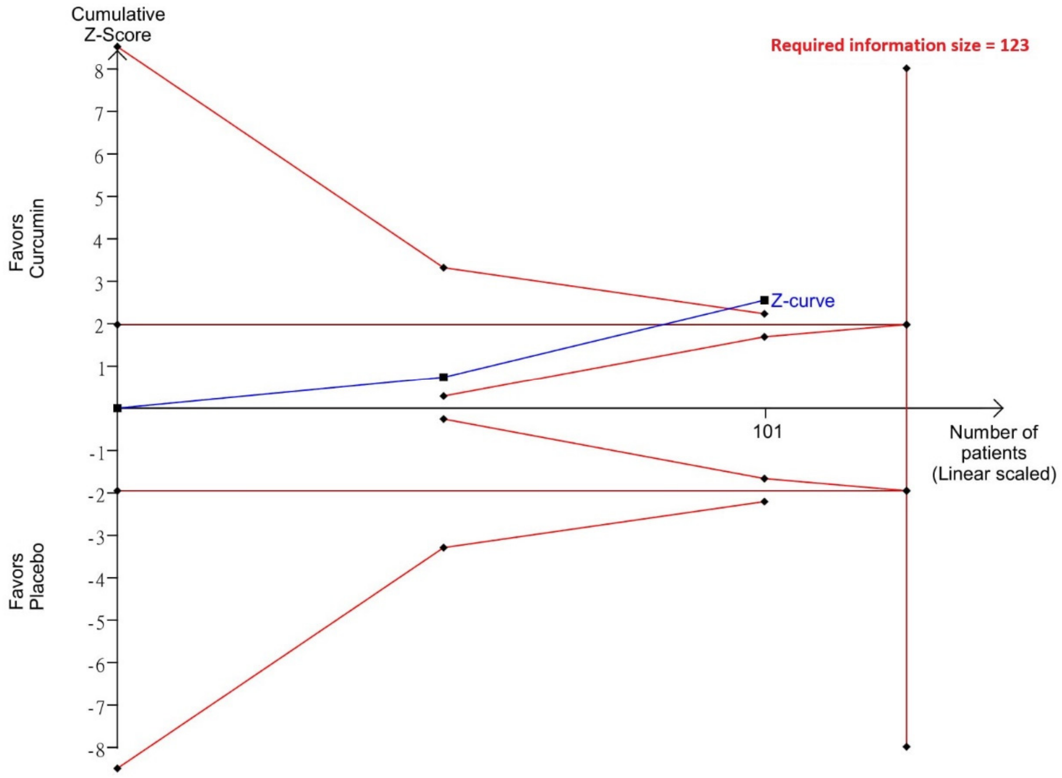

Supplementary Figure S7. Trial sequential analysis of total cholesterol.

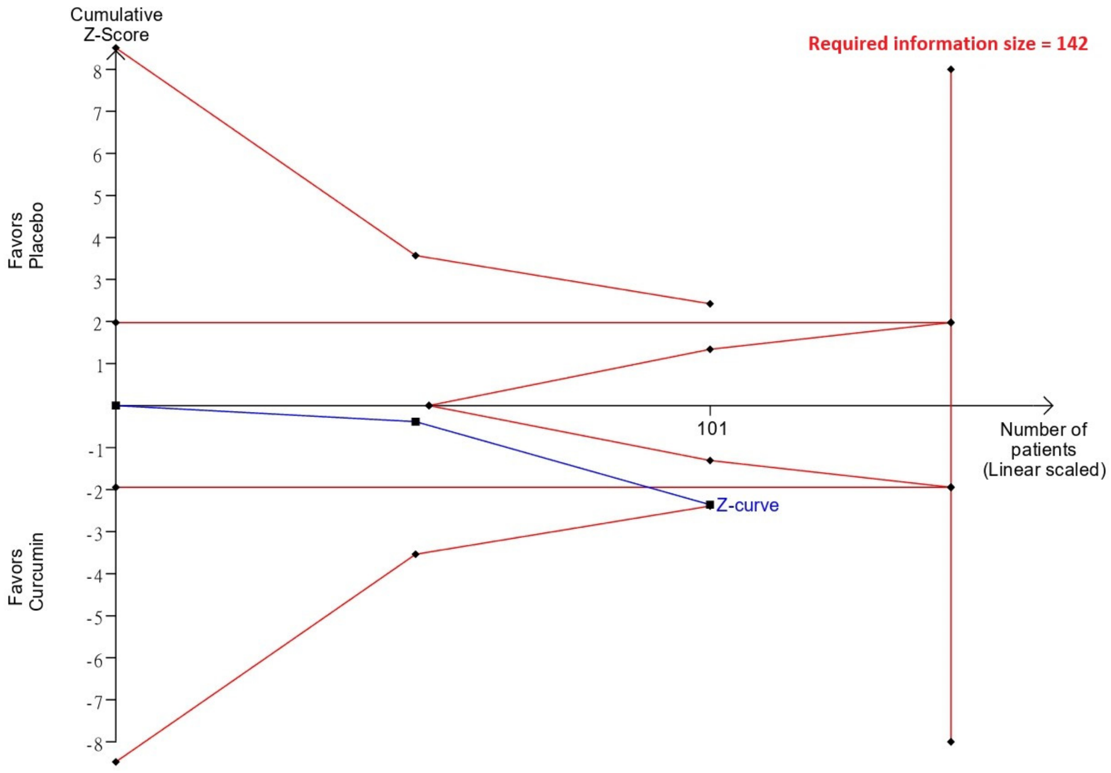

Supplementary Figure S8. Trial sequential analysis of HDL.

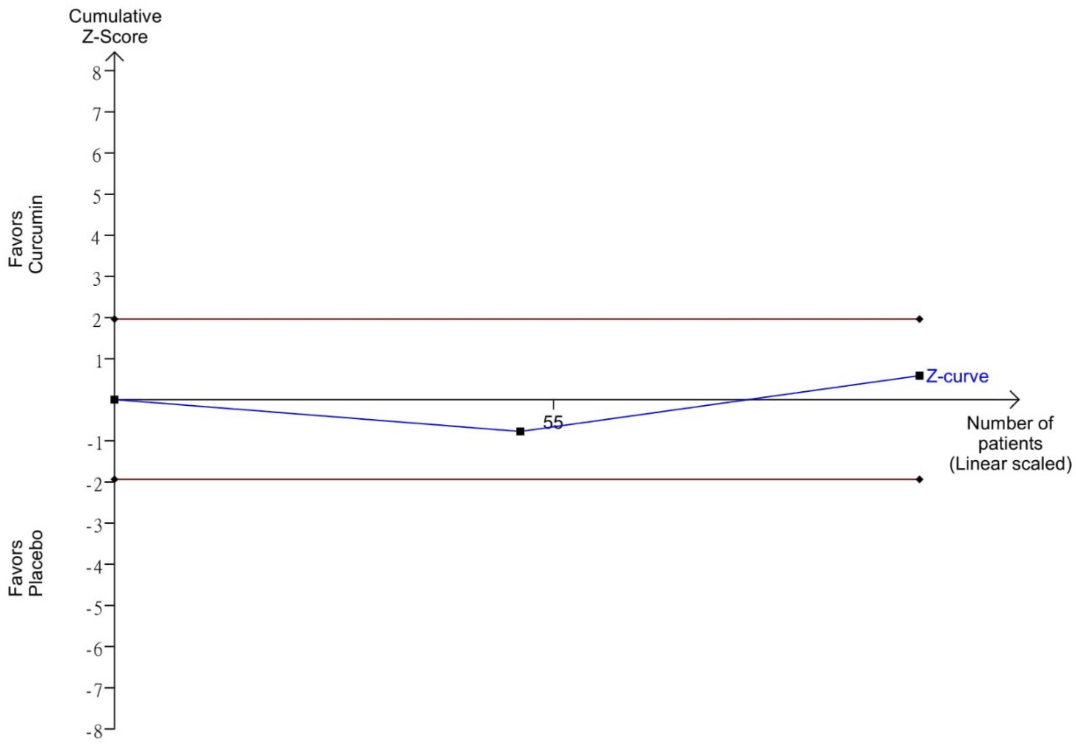

Supplementary Figure S9. Trial sequential analysis of LDL.

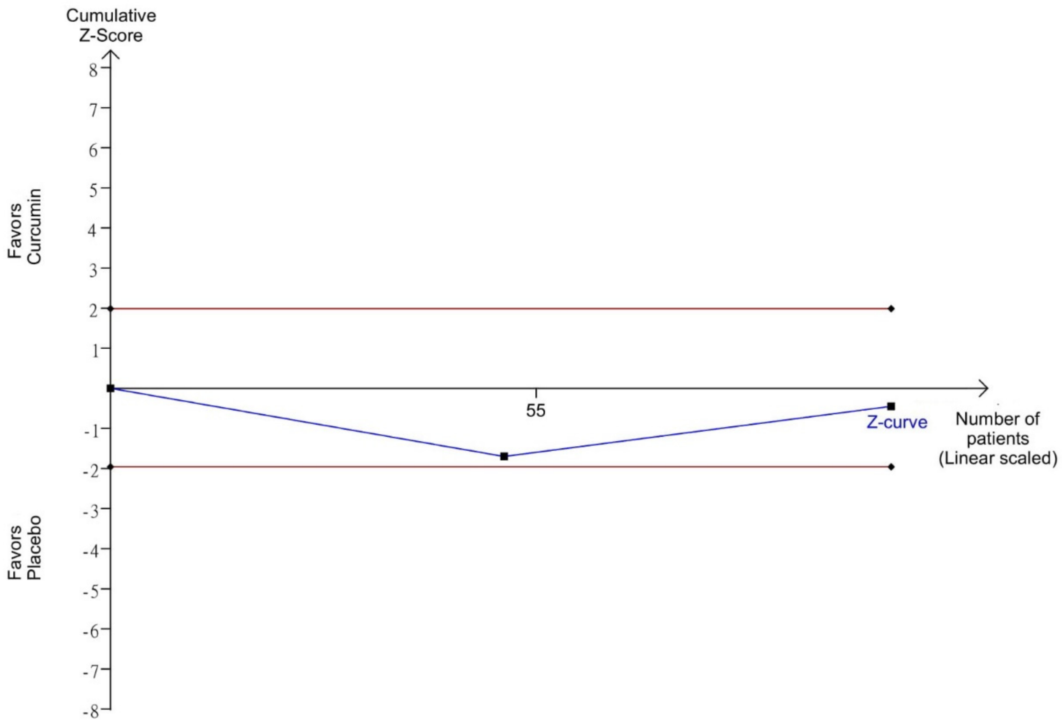

Supplementary Figure S10. Trial sequential analysis of triglyceride.
